# Effectiveness of acupuncture for post-stroke aphasia: protocol for a systematic review and meta-analysis

**DOI:** 10.3389/fneur.2025.1614868

**Published:** 2025-07-25

**Authors:** Junzhe Jia, Yuanli Shan, Yihe Tang, Qiang Tang

**Affiliations:** ^1^Heilongjiang University of Traditional Chinese Medicine, Harbin, Heilongjiang, China; ^2^The Second Affiliated Hospital of Heilongjiang University of Traditional Chinese Medicine, Harbin, Heilongjiang, China; ^3^The First Affiliated Hospital of Heilongjiang University of Traditional Chinese Medicine, Harbin, Heilongjiang, China

**Keywords:** acupuncture, post-stroke aphasia, stroke, aphasia, language disorders, overview of systematic reviews, systematic review, meta-analysis

## Abstract

**Background:**

Post-stroke aphasia (PSA) is a prevalent and debilitating consequence of stroke, significantly impairing communication abilities and reducing patients’ quality of life. While speech and language therapy represents standard care, many patients experience incomplete recovery. Acupuncture, a key component of traditional Chinese medicine, offers a potential complementary approach for enhancing language rehabilitation. This protocol describes a planned systematic review and meta-analysis to synthesize evidence on the effectiveness and safety of acupuncture for patients suffering from post-stroke aphasia.

**Methods and analysis:**

We will systematically search PubMed, Embase, Cochrane Central Register of Controlled Trials (CENTRAL), China National Knowledge Infrastructure (CNKI), Wanfang Database, and China Biology Medicine disc (CBM) from their inception through April 2025. Randomized controlled trials (RCTs) including adult patients (age ≥18 years) diagnosed with post-stroke aphasia will be eligible. The primary outcomes will be changes in overall language function (e.g., using the Western Aphasia Battery) and functional communication ability (e.g., using the Communicative Effectiveness Index). Secondary outcomes will include a dichotomized overall effective rate (based on predefined improvement thresholds reported in the primary studies), quality of life, and adverse events. Outcomes will be analyzed at two pre-specified time points: immediately post-treatment (within 4 weeks of the final session) to assess short-term efficacy, and at long-term follow-up (≥3 months post-treatment) to assess the durability of effects. The quality of included trials will be evaluated using the Cochrane risk bias measurement tool: Risk of Bias 2 (RoB 2). Meta-analysis will be performed using standard review software (e.g., RevMan). Advanced analyses, including meta-regression to explore sources of heterogeneity, will be conducted using the R statistical environment. If meta-analysis is inappropriate due to extreme heterogeneity, a narrative synthesis will be conducted.

**Discussion:**

This study will provide a comprehensive systematic review and meta-analysis focused on RCTs evaluating acupuncture for post-stroke aphasia. Publishing this protocol ensures transparency and outlines the methodology rigorously to avoid duplication. The results aim to provide clinicians and researchers with consolidated evidence regarding the role of acupuncture in post-stroke aphasia management and may inform future clinical guidelines and research directions.

## 1 Introduction

Stroke remains a principal cause of mortality and long-term disability globally, imposing substantial economic and social burdens (1). Post-stroke aphasia (PSA), an acquired impairment affecting language comprehension and/or production, represents one of its most functionally limiting sequelae, impacting approximately one-third of stroke survivors (2). Beyond the core language deficits, PSA profoundly disrupts daily communication, frequently leading to social isolation (3), depression (4), diminished quality of life for both patients and caregivers (5), and increased healthcare utilization (6).

Current standard care for PSA primarily involves speech and language therapy (SLT), which aims to restore impaired language functions and facilitate compensatory communication strategies (7, 8). While SLT demonstrably benefits many individuals, therapeutic gains are often variable, recovery plateaus are common, and a significant proportion of patients are left with chronic (9), functionally significant communication impairments (10). This underscores the critical need to explore and validate effective adjuvant therapies capable of augmenting language recovery beyond what is achievable with SLT alone.

Acupuncture, a therapeutic modality rooted in traditional Chinese medicine involving the precise stimulation of specific acupoints (11), is increasingly utilized within stroke rehabilitation paradigms worldwide (12). Its potential therapeutic mechanisms in the context of neurological recovery, including PSA, are hypothesized to involve the modulation of neuroplasticity, enhancement of cerebral perfusion, regulation of neurotransmitter levels, and mitigation of neuroinflammation, supported by emerging preclinical and clinical evidence (13). Various techniques, such as manual acupuncture (MA), electroacupuncture (EA), and scalp acupuncture, have been applied, often adjunctively with SLT (14).

Despite the growing interest and application, the clinical efficacy of acupuncture for PSA remains a subject of ongoing debate (15). Numerous primary studies, predominantly randomized controlled trials (RCTs), have yielded diverse findings (16). While several systematic reviews (SRs) have attempted to synthesize this evidence, their conclusions are often hampered by limitations. These include the inclusion of studies with high risk of bias, inadequate consideration of heterogeneity stemming from variations in acupuncture protocols and control interventions, potentially outdated literature searches failing to capture recent high-quality trials, and insufficient assessment of specific language outcomes beyond global measures (17). Consequently, a methodologically robust and up-to-date SR focusing specifically on high-quality evidence (RCTs) is crucial to provide a clearer assessment of acupuncture’s true effect size and safety profile for PSA.

Therefore, we outline the protocol for a systematic review and meta-analysis of RCTs designed to rigorously evaluate the effectiveness and safety of acupuncture (compared to sham acupuncture, no treatment, standard care including SLT, or other active therapies) for improving language function, functional communication, and quality of life in patients with post-stroke aphasia. This protocol adheres to the Preferred Reporting Items for Systematic reviews and Meta-Analyses (PRISMA) guidelines (18) to ensure transparency and methodological rigor.

## 2 Methods

### 2.1 Study registration

This review protocol is registered in the International Prospective Register of Systematic Reviews (PROSPERO) as CRD420251033454. Reporting standard followed the Preferred Reporting Items for Systematic Reviews and Meta-Analyses (PRISMA) protocols (see Supplementary material).

### 2.2 Eligibility criteria for study inclusion

#### 2.2.1 Type of study

Eligible studies are limited to RCTs evaluating acupuncture for post-stroke aphasia, defined by formal random allocation methods (parallel-group or cluster designs acceptable).

Studies using quasi-randomization, non-randomized controlled trials (NRCTs), uncontrolled trials, observational studies (cohort, case–control), case reports/series, and preclinical studies will be excluded.

Other systematic reviews and meta-analyses will not be included in our data synthesis to prevent evidence duplication, but their reference lists will be screened for potentially eligible primary RCTs. While full publications are preferred, conference abstracts reporting sufficient data from unpublished eligible RCTs may be considered.

This restriction to RCTs aims to minimize selection bias and ensure the highest level of evidence for assessing treatment effectiveness.

#### 2.2.2 Type of intervention

Eligible interventions must involve invasive acupuncture (needle insertion) targeting post-stroke aphasia. This includes various techniques such as MA, EA, Scalp Acupuncture, and Auricular Acupuncture. Acupuncture may be applied standalone or as an adjunct to other therapies (e.g., speech and language therapy [SLT]), provided its specific effect can be compared to the control group.

There are no restrictions on specific acupoints, stimulation parameters, or treatment schedules for inclusion; these details will be recorded during data extraction.

Studies evaluating non-invasive acupoint stimulation (e.g., acupressure, laser acupuncture) or complex Traditional Chinese Medicine (TCM) interventions where the specific effect of acupuncture cannot be isolated will be excluded. Moxibustion or TENS applied to acupoints will also be excluded unless used solely as a comparator or their effects are clearly separable from needle acupuncture.

#### 2.2.3 Type of controls

Eligible RCTs must compare acupuncture (alone or as an adjunct) against at least one of the following non-acupuncture control conditions:

Sham or placebo acupuncture: To rigorously control for the non-specific effects of the needling procedure and to ensure the quality of blinding, eligible sham or placebo acupuncture controls must be clearly defined and operationalized. We will prioritize RCTs that utilize high-quality sham designs, which must meet at least one of the following criteria:

- Non-penetrating sham devices: The control group receives simulated acupuncture using a validated, non-penetrating placebo needle (e.g., Streitberger or Park needle). This device mimics the sensation of needling by pricking the skin without piercing it and is applied at the same acupoints as the treatment group. This is considered a high-quality method for maintaining patient blinding.
- Non-acupoint stimulation: The control group receives true needle insertion at locations distant from classical acupoints or major nerve pathways, which are considered therapeutically inert for post-stroke aphasia according to traditional Chinese medicine theory or have been validated as such. The rationale for the selection of these non-acupoint locations must be described in the study.

Studies using other forms of sham, such as minimal or superficial needling at true acupoints, will be categorized separately. Their potential physiological activity will be noted, and they will be included in a pre-specified sensitivity analysis to assess their impact on the overall effect estimate. During data extraction, we will systematically record the specific details of the sham intervention used in each study, in accordance with the STRICTA (Standards for Reporting Interventions in Clinical Trials of Acupuncture) guidelines.

No treatment or waiting list control: Where the control group receives no specific intervention for aphasia or is waitlisted.

Standard care/conventional therapy: Including usual rehabilitation or specific treatments like speech and language therapy (SLT). Studies comparing standard care plus acupuncture versus standard care alone are eligible.

Other active therapies: Comparisons against different active interventions targeting post-stroke aphasia.

Studies that only compare different types or parameters of acupuncture against each other, without one of the above non-acupuncture control groups, will be excluded. Studies without any concurrent control group will also be excluded.

#### 2.2.4 Type of outcome measure

Eligible RCTs must report at least one relevant outcome measure assessing acupuncture’s effectiveness or safety for post-stroke aphasia. Primary outcomes of interest are overall language function measured by standardized comprehensive batteries such as the Western Aphasia Battery-Aphasia Quotient (WAB-AQ) or the Boston Diagnostic Aphasia Examination (BDAE) Severity Rating; functional communication ability assessed through validated scales reflecting real-world communication like the Communicative Effectiveness Index (CETI) or the ASHA Functional Assessment of Communication Skills for Adults (ASHA-FACS); and the overall effective rate. This dichotomous outcome is frequently reported in the primary literature and is typically defined based on a pre-specified threshold of improvement on a validated language scale. For this review, the overall effective rate will be defined as the proportion of participants who meet the explicit criteria for “effective” as defined in each primary study. These criteria are commonly based on:

- A specific percentage improvement from baseline on a continuous scale (e.g., an increase of >30% in the WAB-AQ score); or.
- An improvement of one or more grades on a clinician-rated ordinal severity scale (e.g., improving from “moderately severe” to “mild”).

During data extraction, we will systematically record the specific definition and threshold used by each study. For the meta-analysis, we will pool the number of participants classified as ‘effective’ in each group. We recognize that definitions may vary and plan to conduct a subgroup analysis based on the type of criteria used (e.g., percentage-based vs. grade-based) if sufficient data are available, to investigate this as a potential source of heterogeneity.

Secondary outcomes encompass improvements in specific language modalities including auditory comprehension, verbal expression, or naming assessed by tools like the Boston Naming Test (BNT); patient-reported quality of life (QoL) using generic or specific validated instruments like EQ-5D or the Stroke and Aphasia Quality of Life Scale-39 (SAQOL-39); and the reporting of adverse events detailing incidence, type, and severity. Studies using different validated measurement instruments for these constructs are acceptable.

To ensure the comparability of data across studies and to distinguish between short-term efficacy and the long-term durability of effects, outcomes will be analyzed at two distinct, pre-specified time points. Studies must report outcome data for at least one of these windows to be included in the relevant meta-analysis:

Immediate post-treatment effect: This is defined as the first outcome assessment conducted immediately after, or within 4 weeks of, the final treatment session. This time point is intended to capture the primary efficacy of the intervention.

Long-term follow-up effect: This is defined as the outcome assessment conducted at 3 months or later (≥3 months) after the cessation of treatment. This time point is intended to evaluate the sustainability of any observed benefits. If a study reports multiple follow-up assessments, we will use the data from the time point closest to 6 months for our primary follow-up analysis to enhance consistency.

Quantitative reporting of outcomes at these specific time points is required for inclusion in the meta-analysis.

#### 2.2.5 Type of exclusion criteria

NRCTs, quasi-RCTs, or other ineligible study designs.

Studies not involving adults (≥18 years) with aphasia secondary to stroke.

Studies evaluating non-invasive acupuncture, inseparable complex interventions, or lacking an eligible non-acupuncture control group.

Studies not reporting quantifiable data for pre-defined relevant outcomes.

Duplicate publications of the same study cohort.

Abstracts, letters, or editorials lacking sufficient data for analysis.

Studies with critical methodological flaws or unresolvable data errors.

### 2.3 Search methods for identification of studies

#### 2.3.1 Electronic data sources

A comprehensive electronic literature search will be conducted to identify relevant RCTs. The following databases will be systematically searched: PubMed, Embase, Cochrane Central Register of Controlled Trials (CENTRAL), China National Knowledge Infrastructure (CNKI), Wanfang Database, and China Biology Medicine disc (CBM). The search will cover the period from the inception of each database through April 2025. No language restrictions will be applied during the search phase. Search strategies will be developed using a combination of Medical Subject Headings (MeSH) or equivalent thesaurus terms and relevant keywords related to stroke, aphasia, and acupuncture.

#### 2.3.2 Searching other resources

Any eligible studies potentially overlooked will also be manually identified by scanning the reference lists of related systematic reviews and conference proceedings.

#### 2.3.3 Search strategy

See Supplementary material for details.

### 2.4 Data collection and analysis

#### 2.4.1 Selection of studies

**Figure 1:**
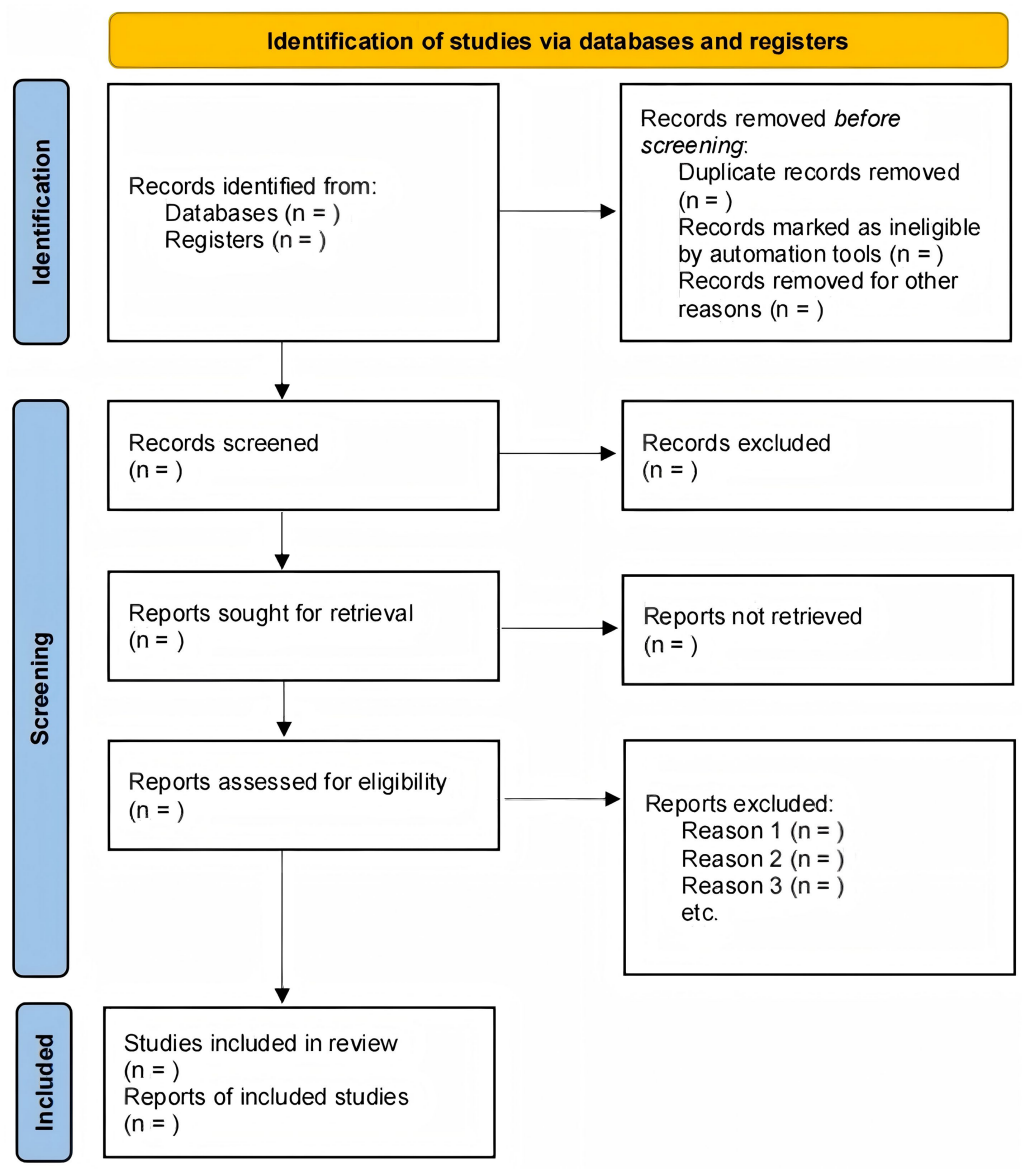
PRISMA flow diagram of Figure 1. The study selection process.

After gathering search results from all databases, duplicates will be identified and removed using reference management software. Subsequently, two independent reviewers (JJ and YS) will screen the titles and abstracts of the remaining records. Studies clearly not meeting the eligibility criteria based on title and abstract will be excluded. The full texts of potentially relevant studies will then be retrieved and independently assessed in detail by the same two reviewers against the pre-defined eligibility criteria. Any disagreements regarding study inclusion at either the screening or full-text assessment stage will be resolved through discussion or, if consensus cannot be reached, by consulting a third reviewer (YT). A Preferred Reporting Items for Systematic reviews and Meta-Analyses (PRISMA) flow diagram will be generated to illustrate the entire study selection process (Figure 1).

#### 2.4.2 Data extraction and management

Two reviewers (JJ and YS) will independently extract data from the included RCTs using a pre-designed and piloted data extraction form, likely developed in Microsoft Excel or directly within review software. This form will capture key information including:

Study characteristics: First author, publication year, country, study design specifics, funding sources.

Participant characteristics: Sample size (total and per group), age, sex, stroke details (type, location, time since onset), baseline aphasia characteristics (type, severity, assessment tool), key inclusion/exclusion criteria.

Intervention details: Type of acupuncture (MA, EA, scalp, etc.), specific acupoints used, stimulation parameters (duration, frequency, intensity), details of needling technique, treatment frequency and total duration, concurrent therapies received by the intervention group (19).

Comparator details: Type of control intervention (sham, standard care, etc.), specifics of the control procedure, concurrent therapies received by the control group.

Outcome measures: All relevant primary and secondary outcomes as defined in Section 2.2.4, measurement tools used, time points of assessment (baseline, end-of-treatment, follow-up).

Results: Quantitative data for each outcome measure at each time point, reported as means and standard deviations (SDs) for continuous outcomes, or number of events and total participants for dichotomous outcomes. Effect estimates (e.g., mean differences, risk ratios) with confidence intervals (CIs) reported by the study will also be extracted.

Adverse events: Type, frequency, and severity reported in each group.

Risk of bias information: Data points relevant to assessing each domain of the RoB 2 tool.

Discrepancies in extracted data between the two reviewers will be resolved by discussion and consensus, or by consultation with a third reviewer (YT or QT) if needed. If essential data are missing or reported unclearly in the publications, we will attempt to contact the corresponding authors for clarification. Extracted data will be managed within systematic review software or a dedicated spreadsheet.

#### 2.4.3 Assessment of risk of bias in included studies

Two reviewers (JJ and YS) will independently assess the risk of bias for each included RCT using the Cochrane Risk of Bias tool, version 2 (RoB 2). Any disagreements will be resolved through discussion or consultation with a third reviewer (YT or QT).

In our assessment, we will place special emphasis on Domain 3: ‘Bias in measurement of the outcome’. Acknowledging that blinding of practitioners is often not feasible in acupuncture trials, the risk of detection bias is a primary concern. Therefore, the implementation and reporting of outcome assessor blinding will be a critical factor in our quality judgment. This specific assessment will directly inform the key sensitivity analysis designed to quantify the impact of this potential bias, as detailed in Section 2.4.9. The results of the risk of bias assessment will be summarized in graphs and tables.

#### 2.4.4 Data synthesis

We will use a two-stage approach for our data synthesis. Primary meta-analyses for pooling effect sizes will be conducted using Review Manager (RevMan, version 5.4), as it is standard for Cochrane reviews and produces clear forest plots.

However, to robustly investigate sources of heterogeneity and formally test for differences between subgroups as outlined in section 2.4.8, we will conduct advanced analyses such as meta-regression and interaction tests using the metafor package in the R statistical environment (version 4.0 or later). This approach is chosen because R offers superior flexibility and power for these more complex statistical models compared to RevMan. Heterogeneity will be assessed using the Chi-squared test and the I^2^ statistic.

#### 2.4.5 Dealing with missing data

We will attempt to contact study authors to retrieve missing data or clarify reported information when necessary. For missing summary statistics required for meta-analysis, we will calculate them from other available statistics if possible, or estimate them using established statistical methods, citing the method used. If crucial data for a specific outcome cannot be obtained or reliably estimated, the study will be excluded from the meta-analysis for that particular outcome but included in the narrative synthesis. The extent and potential impact of missing participant outcome data (attrition) will be assessed within the ‘Bias due to missing outcome data’ domain of the RoB 2 tool. Sensitivity analyses may be performed to explore the impact of different assumptions about missing data if deemed necessary and feasible.

#### 2.4.6 Assessment of heterogeneity

Statistical heterogeneity between studies included in a meta-analysis will be assessed to determine the appropriateness of pooling data and to inform the choice of model. We will use the Chi-squared (*χ*^2^) test, with a *p*-value < 0.10 considered indicative of statistically significant heterogeneity. The degree of heterogeneity will be quantified using the I^2^ statistic, where values roughly representing low (<25%), moderate (25–75%), and high (>75%) heterogeneity will be considered, although interpretation will also depend on the context and number of studies. Visual inspection of forest plots will also be performed to aid in identifying heterogeneity patterns. Significant heterogeneity (e.g., I^2^ > 50–75% or significant *χ*^2^) will primarily lead to the use of a random-effects model for pooling and may prompt further investigation through subgroup or sensitivity analyses, or potentially indicate that meta-analysis is inappropriate for that specific outcome.

#### 2.4.7 Assessment of publication biases

Assessment of potential publication bias will be conducted if a meta-analysis for a specific outcome includes at least 10 studies. We will visually inspect funnel plots, plotting study effect estimates against a measure of precision. Asymmetry in the funnel plot may suggest publication bias, although heterogeneity or other factors (such as methodological quality) can also be explanations. Formal statistical testing for funnel plot asymmetry, such as Egger’s regression test, may be used as an adjunct to visual inspection where appropriate. Interpretation will be performed with caution, acknowledging the limitations of these methods, particularly with small numbers of studies or substantial heterogeneity.

#### 2.4.8 Subgroup analysis

To systematically investigate anticipated sources of clinical and methodological heterogeneity, we will conduct several mandatory, pre-specified subgroup analyses, provided sufficient data (at least two studies per subgroup) are available. These analyses are critical for understanding potential treatment effect moderators:

Acupuncture technique: We will separately analyze studies based on the primary acupuncture intervention used: (a) Manual Acupuncture (MA), (b) Electroacupuncture (EA), and (c) Scalp Acupuncture. This is crucial as these techniques may have distinct neurophysiological mechanisms and clinical efficacy.

Time since stroke onset: We will stratify studies according to the patient’s recovery phase, defined as: (a) Acute phase (<1 month post-stroke), (b) Subacute phase (1 to 6 months post-stroke), and (c) Chronic phase (>6 months post-stroke). This is to account for the significant influence of spontaneous recovery and differing neuroplastic potential across these stages.

Intensity of concurrent speech and language therapy (SLT): Recognizing that SLT is the standard of care, we will analyze subgroups based on the intensity of SLT provided to both the intervention and control groups (e.g., high-intensity SLT vs. low-intensity/unspecified SLT vs. no SLT). This will help determine if acupuncture acts as a standalone therapy or as an adjuvant whose effectiveness is dependent on the intensity of conventional rehabilitation.

Additional exploratory subgroup analyses will be conducted to further explore sources of variability, based on: (a) definition of the overall effective rate (e.g., percentage-based vs. grade-based criteria), (b) control type (sham vs. standard care), (c) baseline aphasia severity (e.g., severe vs. mild–moderate), and (d) overall risk of bias (low vs. high/some concerns). We will use formal statistical tests for interaction (*p* < 0.10) to assess for significant differences between subgroups. Given the observational nature of subgroup comparisons, all findings will be interpreted with caution as hypothesis-generating rather than confirmatory.

#### 2.4.9 Sensitivity analysis

To assess the robustness of our pooled findings, we will conduct the following pre-specified sensitivity analyses:

A critical sensitivity analysis to address bias inherent in acupuncture trials. Given that performance bias (due to the inability to blind practitioners) is unavoidable, the potential for detection bias is high. To specifically evaluate its impact, we will conduct a sensitivity analysis by repeating the primary meta-analysis including only those studies that implemented adequate blinding of outcome assessors (i.e., studies judged as having a ‘Low risk of bias’ in the ‘Bias in measurement of the outcome’ domain of the RoB 2 tool). We will compare this result with the main analysis to determine if a lack of assessor blinding significantly influenced the overall effect estimate.

Excluding studies with an overall ‘High risk’ of bias to evaluate the impact of lower-quality evidence on the summary effect.

Changing the statistical model (e.g., from a random-effects to a fixed-effect model) to check the stability of the result.

We will examine whether the direction and magnitude of the effect estimate remain stable under these different analytical assumptions.

### 2.5 Grading the quality of evidence

Two reviewers (JJ and YS) will independently assess the overall quality of evidence for major outcomes using the Grading of Recommendations Assessment, Development and Evaluation (GRADE) approach. We will evaluate potential downgrading factors including risk of bias, inconsistency, indirectness, imprecision, and publication bias. Based on these assessments, the evidence for each outcome will be rated as High, Moderate, Low, or Very Low. Disagreements will be resolved by discussion or a third reviewer (YT or QT). The final quality ratings and supporting evidence will be presented in a Summary of Findings (SoF) table.

### 2.6 Patient and public involvement

There was no patient or public involvement in the development of this systematic review protocol.

### 2.7 Dissemination and ethics

The findings of this systematic review and meta-analysis will be disseminated through publication in a relevant peer-reviewed journal. We also intend to present the results at national or international scientific conferences related to stroke rehabilitation, acupuncture, or evidence-based medicine.

As this study is a systematic review based entirely on previously published and publicly available data from other studies, formal ethical approval from an institutional review board or ethics committee is not required. All data used will be extracted from the published literature, ensuring no direct involvement of human participants or breach of individual patient confidentiality by the review team. We will operate under the assumption that the primary RCTs included in this review obtained appropriate ethical approvals and participant consent prior to their original conduct.

## 3 Discussion

This systematic review protocol outlines the methodology for a comprehensive evaluation of the effectiveness and safety of acupuncture for treating post-stroke aphasia based on evidence from RCTs. Post-stroke aphasia severely impacts patients’ communication, mental health, and QoL, representing a significant burden for individuals, families, and society. Although SLT is the cornerstone of rehabilitation, recovery of language function remains suboptimal or slow for many patients, highlighting the clinical need to explore effective adjunctive or alternative therapies (20). Acupuncture, a traditional therapy widely used in post-stroke rehabilitation, especially in Asia, requires a systematic and updated assessment of its specific effects and evidence strength for improving aphasia (21).

While the precise mechanisms underlying acupuncture’s effects on post-stroke recovery and aphasia are still under investigation, several pathways have been proposed. These potentially include the modulation of cerebral hemodynamics (22), promotion of neuroplasticity (23), regulation of relevant neurotransmitters, reduction of neuroinflammation in peri-infarct regions (24), and modulation of functional connectivity within brain networks crucial for language processing. Furthermore, different acupuncture techniques included in this review might exert effects through distinct pathways or to varying degrees. Indeed, several individual RCTs and previous preliminary reviews have suggested potential benefits of acupuncture for improving various aspects of language function post-stroke (25), such as overall language scores or specific modality performance (26). However, findings have sometimes been inconsistent, and methodological limitations in primary studies often cloud the interpretation. Therefore, a key aim of this systematic review is to rigorously synthesize the existing evidence regarding these potential benefits. However, in synthesizing this evidence, it will be crucial to interpret the findings with caution. A significant portion of the primary research is expected to originate from China, and we must remain cognizant of the potential for language and location-specific biases that may favor positive outcomes, an issue we will address explicitly in our analysis and limitations.

Strengths of this planned review include a comprehensive search strategy across major international and Chinese databases without language restrictions, minimization of bias through duplicate independent screening and data extraction, use of the validated Cochrane RoB 2 tool for risk of bias assessment (27), and application of the GRADE approach to rate the certainty of the evidence (28). This systematic methodology is designed to ensure the reliability and transparency of our findings.

While we acknowledge the inherent challenges in synthesizing evidence in this field, particularly concerning study quality and variability, our pre-specified analysis plan is designed to address these issues rigorously. Through careful assessment of heterogeneity, appropriate use of statistical models, planned subgroup analyses, and reliance on narrative synthesis when meta-analysis is inappropriate, we aim to provide a transparent interpretation of the available evidence.

The potential implications of this review are significant. If the evidence indicates that acupuncture is effective and safe, our findings could inform clinical practice guidelines and support the integration of acupuncture into multidisciplinary post-stroke rehabilitation programs. Conversely, if the evidence is insufficient, of low quality, or suggests limited benefit, this review will underscore the need for caution in clinical application and, importantly, guide future research by identifying methodological shortcomings in existing trials and suggesting priorities for higher-quality RCTs. The GRADE-based SoF table will provide clinicians, patients, and policymakers with a clear overview of the current evidence base and the confidence we can place in it, facilitating informed decision-making regarding the use of acupuncture alongside standard care (29).

In conclusion, this systematic review aims to provide an up-to-date and rigorous synthesis of RCT evidence on acupuncture for post-stroke aphasia, ultimately intending to inform clinical decisions and direct future research in this field.

### 3.1 Innovation points

This study protocol aims to offer distinct value beyond existing literature through several key aspects. Firstly, by employing a rigorous bilingual search strategy integrating major international and Chinese databases, we strive to comprehensively capture the global research evidence, aiming to mitigate potential publication and language biases—a breadth potentially unattained by many single-language reviews. Secondly, building on this comprehensive dataset, we plan an in-depth exploration of statistical heterogeneity. Beyond simply assessing its presence (using I^2^ and Tau^2^), we will critically explore its potential sources through pre-specified, clinically relevant subgroup analyses. This detailed approach is intended to guide a nuanced interpretation and potentially provide more targeted conclusions than reviews simply reporting a single pooled effect estimate. Furthermore, this review will sharpen the focus on potential differences in efficacy between various acupuncture interventions and systematically evaluate the impact on patient-reported outcomes of significant clinical importance, such as functional communication ability and QoL—aspects potentially underemphasized in prior research. Therefore, the innovation lies primarily in the synergy of comprehensive evidence retrieval, deep heterogeneity management, and a specific focus on distinct intervention types and patient-important outcomes. This approach aims to deliver a systematic review that is not only methodologically robust but also possesses stronger practical utility in terms of clinical relevance and guidance.

### 3.2 Limitations

We anticipate several potential limitations in this systematic review. Firstly, the methodological quality of included RCTs may vary considerably, with potential risks of bias related to randomization, allocation concealment, blinding (especially challenging for acupuncture interventions and outcome assessment), and handling of missing data; poor quality studies could affect the validity of our findings. Additionally, significant clinical and statistical heterogeneity is expected due to variations across studies in participant characteristics (e.g., stroke type, aphasia severity, time since onset), acupuncture protocols (e.g., techniques, acupoints, treatment schedules), comparator types, and outcome measures used. Such heterogeneity might limit the appropriateness of meta-analysis for some outcomes or necessitate cautious interpretation of pooled results. Furthermore, our review is susceptible to publication and language bias. There is a general concern that studies with statistically significant findings are more likely to be published. More specifically, given that acupuncture is a therapy originating from and widely practiced in China, a substantial proportion of the evidence is expected to come from Chinese trials published in their native language. It has been noted that studies from China may have a higher tendency to report positive findings for acupuncture, potentially introducing a language or location bias that could lead to an overestimation of the treatment effect. While our bilingual search strategy is designed to retrieve this literature comprehensively, it does not mitigate any inherent bias within that body of evidence. We will use funnel plot assessment and Egger’s test to explore small-study effects, but we acknowledge these methods cannot definitively distinguish between publication bias, language bias, and true clinical heterogeneity.

Poor reporting quality in primary studies might also be an issue, potentially hindering detailed analysis, particularly regarding intervention parameters or specific outcome data. Finally, while our search strategy is designed to be comprehensive, we may inevitably miss some relevant studies, particularly unpublished ones or those in less accessible grey literature. Although our search includes major Chinese databases and has no language restrictions, potential challenges in accurately extracting or interpreting data from studies in languages other than English or Chinese could represent a residual limitation. These anticipated limitations will be carefully considered when interpreting the review’s findings and formulating conclusions.

## Glossary

IS: ischemic stroke
PSA: post-stroke aphasia
RCT: Randomized Controlled Trial
SLT: speech and language therapy
MA: manual acupuncture
EA: electroacupuncture
WAB-AQ: Western Aphasia Battery-Aphasia Quotient
BDAE: Boston Diagnostic Aphasia Examination
CETI: Communicative Effectiveness Index
SAQOL-39: Stroke and Aphasia Quality of Life Scale-39
RoB 2: Cochrane Risk of Bias Tool for Randomized Trials, version 2
PRISMA: Preferred Reporting Items for Systematic reviews and Meta-Analyses
MD: mean difference
SMD: standardized mean difference
RR: risk ratio
OR: odds ratio
CI: confidence interval
CNKI: China National Knowledge Infrastructure
CBM: China Biology Medicine Disc
CENTRAL: Cochrane Central Register of Controlled Trials
