# Supplementary material for: Effectiveness of acupuncture for post-stroke aphasia: protocol for a systematic review and meta-analysis
Source: Front Neurol. 2025 Jul 25;16:1614868. doi: 10.3389/fneur.2025.1614868 (PMC12331734; doi:10.3389/fneur.2025.1614868)
Supplement: Supplementary file 1 [file Data_Sheet_1.pdf]

Database	No	Search Terms
China National Knowledge Infrastructure (CNKI)	1	TKA%=('中风' + '脑卒中' + '脑血管意外' + '脑梗死' + '脑出血') AND TKA%=('失语' + '语言障碍') AND TKA%=('针刺' + '针灸' + '电针' + '头皮针' + '体针') AND TKA%=('随机' + '对照' + '临床试验' + '分组') NOT TKA%=('动物' + '鼠' + '兔' + '综述' + 'Meta 分析')
Wanfang Database	1	主题:(中风 or 脑卒中 or 脑梗死 or 脑出血) AND 主题:(失语 or 语言障碍) AND 主题:(针刺 or 针灸 or 电针 or 头皮针) AND 主题:(随机 or 对照 or 临床试验 or 分组) NOT 主题:(动物 or 鼠 or 兔 or 综述 or Meta 分析)
VIP Database (Chongqing VIP)	1	篇关摘：中风 or 脑卒中 or 脑梗死 or 脑出血
	2	篇关摘：失语 or 语言障碍
	3	篇关摘：针刺 or 针灸 or 电针 or 头皮针 or 体针
	4	篇关摘：随机 or 对照 or 临床试验 or 分组
	5	篇关摘：动物 or 鼠 or 兔 or 综述 or Meta 分析
	6	#1 AND #2 AND #3 AND #4 NOT #5
China Biology Medicine disc (CBM)	1	('中风'[常用字段:智能] OR '脑卒中'[常用字段:智能] OR '脑梗死'[常用字段:智能] OR '脑出血'[常用字段:智能]) AND ('失语'[常用字段:智能] OR '语言障碍'[常用字段:智能]) AND ('针刺'[常用字段:智能] OR '针灸'[常用字段:智能] OR '电针'[常用字段:智能] OR '头皮针'[常用字段:智能]) 限定条件： 随机对照试验; 对照试验; 随机化; (Meta 分析[文献类型] OR 综述[文献类型]) NOT
Web of Science (Core Collection)	1	(TS=(stroke OR cerebrovascular accident OR CVA OR cerebral infarction OR cerebral hemorrhage)) AND (TS=(aphasia OR dysphasia OR language disorder* OR speech disorder*)) AND (TS=(acupuncture OR electroacupuncture OR EA OR manual acupuncture OR MA OR scalp acupuncture OR needling)) AND (TS=(randomized controlled trial OR controlled clinical trial OR randomized OR randomly OR trial OR groups)) NOT TS=(animal* OR rat OR rats OR mouse OR mice OR rabbit*)
Embase	1	('stroke'/exp OR 'cerebrovascular accident'/exp OR 'cerebral infarction'/exp OR 'brain hemorrhage'/exp OR stroke:ti,ab,kw OR cva:ti,ab,kw)
	2	('aphasia'/exp OR 'dysphasia'/exp OR 'language disorder'/exp OR 'speech disorder'/exp OR aphasia:ti,ab,kw OR dysphasia:ti,ab,kw)
	3	('acupuncture'/exp OR 'electroacupuncture'/exp OR 'scalp acupuncture':ti,ab,kw OR 'manual acupuncture':ti,ab,kw OR needling:ti,ab,kw)
	4	('randomized controlled trial'/exp OR 'controlled clinical trial'/exp OR random*:ti,ab,kw OR trial:ti,ab,kw OR groups:ti,ab,kw)
	5	('animal'/exp NOT 'human'/exp) OR animal*:ti,ab,kw OR rat:ti,ab,kw OR mouse:ti,ab,kw OR rabbit:ti,ab,kw
	6	#1 AND #2 AND #3 AND #4 NOT #5
Cochrane Central Register of	1	([stroke] OR [cerebrovascular accident] OR [cerebral infarction] OR [brain hemorrhage] OR stroke:ti,ab,kw OR cva:ti,ab,kw)

Controlled Trials (CENTRAL)	2	([aphasia] OR [dysphasia] OR [language disorder] OR [speech disorder] OR aphasia:ti,ab,kw OR dysphasia:ti,ab,kw)
	3	([acupuncture therapy] OR [electroacupuncture] OR acupuncture:ti,ab,kw OR electroacupuncture:ti,ab,kw OR "scalp acupuncture":ti,ab,kw OR needling:ti,ab,kw)
	4	#1 AND #2 AND #3
PubMed	1	(Stroke[mh] OR Cerebrovascular Accident[mh] OR Cerebral Infarction[mh] OR Cerebral Hemorrhage[mh] OR stroke[tiab] OR CVA[tiab])
	2	(Aphasia[mh] OR Language Disorders[mh] OR Speech Disorders[mh] OR aphasia[tiab] OR dysphasia[tiab])
	3	(Acupuncture Therapy[mh] OR Electroacupuncture[mh] OR acupuncture[tiab] OR electroacupuncture[tiab] OR "scalp acupuncture"[tiab] OR needling[tiab])
	4	(Randomized Controlled Trial[pt] OR Controlled Clinical Trial[pt] OR randomized[tiab] OR randomly[tiab] OR trial[tiab] OR groups[tiab])
	5	#1 AND #2 AND #3 AND #4 NOT (animals[mh] NOT humans[mh])
Chictr	1	检索词 (主题或研究题目): (中风 OR 卒中) AND (失语 OR 语言障碍) AND (针刺 OR 针灸 OR 电针 OR 头针) 研究类型: 干预性研究 OR 治疗研究
ClinicalTrials.gov	1	Condition or disease: (Stroke OR Cerebrovascular Accident) AND (Aphasia OR Language Disorder) Intervention/treatment: Acupuncture OR Electroacupuncture OR Needling OR Scalp Acupuncture Study Type: Interventional Studies (Clinical Trials)
OpenGrey	1	(stroke OR aphasia) AND (acupuncture OR needling) AND (randomized OR trial OR controlled)
WorldCat	1	Subject=(stroke OR cerebrovascular accident) AND Subject=(aphasia OR language disorder)
	2	Subject=(acupuncture OR electroacupuncture OR needling)
	3	Subject=(randomized controlled trial OR clinical trial OR controlled trial)
	4	#1 AND #2 AND #3
Scopus	1	(TITLE-ABS-KEY=(stroke OR cerebrovascular OR cva OR infarction OR hemorrhage)) AND (TITLE-ABS-KEY=(aphasia OR dysphasia OR "language disorder" OR "speech disorder")) AND (TITLE-ABS-KEY=(acupuncture OR electroacupuncture OR "scalp acupuncture" OR needling)) AND (DOCTYPE(ar) OR DOCTYPE(cp)) AND (LIMIT-TO(EXACTKEYWORD,"Randomized Controlled Trial") OR TITLE-ABS-KEY(randomized OR randomly OR trial OR groups)) AND (LIMIT-TO(SUBJAREA,"MEDI") OR LIMIT-TO(SUBJAREA,"NEUR")) NOT (LIMIT-TO(EXACTKEYWORD,"Animal") OR LIMIT-TO(EXACTKEYWORD,"Rat") OR LIMIT-TO(EXACTKEYWORD,"Mouse"))

CNKI: TKA=title OR keyword OR abstract, %= 'str' indicates that the relevant record matches str.

Web of Science: TS=title OR abstract OR keyword.

Embase: ti=title, ab=abstract, kw=keyword, exp=Emtree term (similar to Mesh term in Pubmed).

Cochrane Central Registry of Controlled Trials (CENTRAL): ti=title, ab=abstract, kw=keyword.

Scopus: TITLE-ABS-KEY=title OR abstract OR keyword.

PubMed: [mh]=MeSH Heading (Medical Subject Headings), [tiab]=title OR abstract, [pt]=publication type.
